# Two-needle-holder endoscopic hand suturing for a mucosal defect after rectal endoscopic submucosal dissection

**DOI:** 10.1055/a-2767-2012

**Published:** 2026-03-16

**Authors:** Junnosuke Hayasaka, Yusuke Kawai, Hitomi Iwasaki, Yorinari Ochiai, Hiroshi Yamato, Akiko Koyama, Shu Hoteya

**Affiliations:** 1 13600 Department of Gastroenterology, Toranomon Hospital, Tokyo, Japan; 2 13600 Endoscopy Division, Toranomon Hospital, Tokyo, Japan

**Fig. 1:**
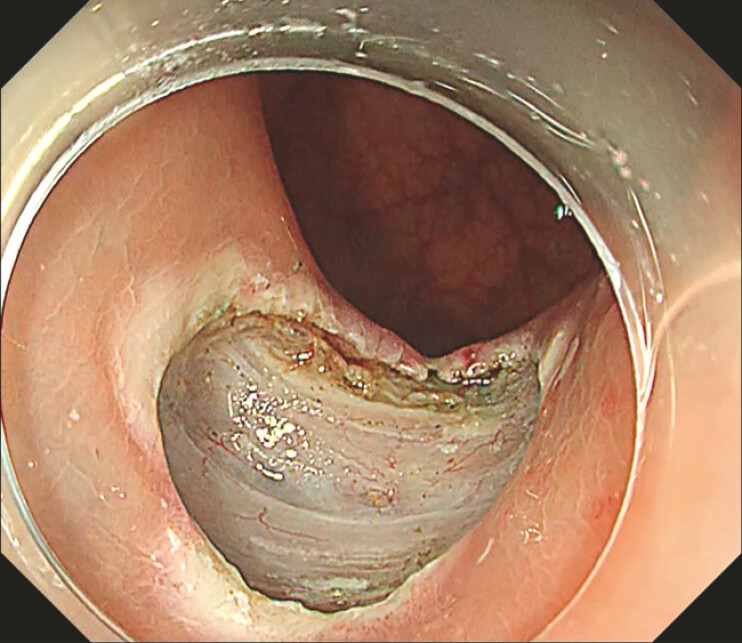
Post-endoscopic submucosal dissection ulcer in the lower rectum (Rb).

**Fig. 2:**
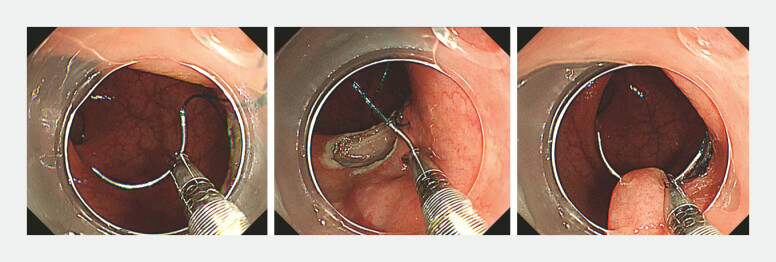
Using the right channel holder, the needle is grasped and inserted into the mucosa; the rotation of the holder externalises the needle tip.

**Fig. 3:**
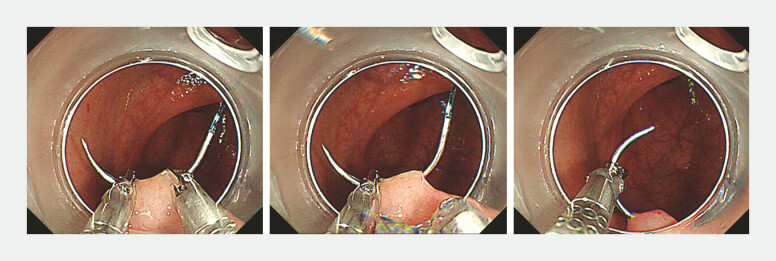
The inserted needle tip is grasped with the left channel holder; after releasing the right holder, the left holder is rotated to withdraw the needle.

**Fig. 4:**
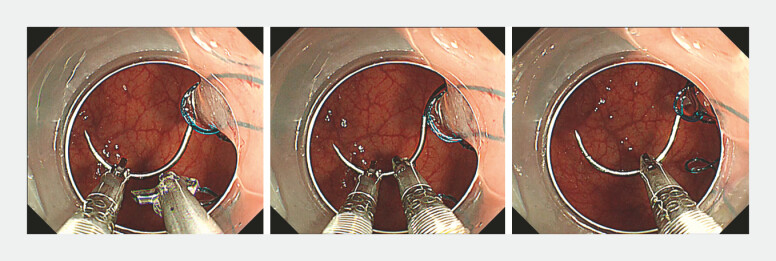
The needle is handed off from the left- to the right-channel holder to continue the running suture.

**Fig. 5:**
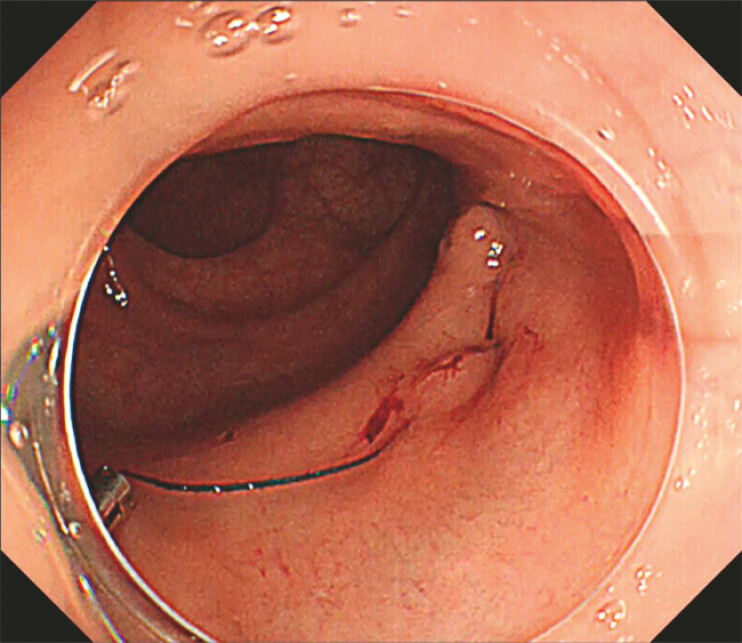
Tightening the suture line completes the closure of the ulcer base.

Endoscopic hand suturing (EHS) is increasingly used to close mucosal defects after gastrointestinal endoscopic submucosal dissection (ESD). EHS has been reported to be safe and technically effective, with evidence suggesting a reduction in post-ESD bleeding 1 2 . Emerging reports have also described EHS after endoscopic full-thickness resection (EFTR 3 4 ). A practical limitation of conventional EHS is its technical difficulty when performed with a single needle holder: the needle must be released and re-grasped repeatedly, creating “free-needle” moments during which the tip can stray. This not only risks destabilising the suture line but also makes re-grasping technically demanding and time-consuming, thereby prolonging the procedure. We present a two-needle-holder EHS ( Video 1 ) that minimises free-needle time and enables stable, continuous suturing. A 68-year-old woman underwent ESD for a 3-mm neuroendocrine tumour in the anterior wall of the lower rectum. Using a double-channel, multi-bending endoscope (GIF-2TQ260M; Olympus), two through-the-scope flexible needle holders (SutuArt FG-260; Olympus), and a barbed absorbable suture (V-Loc 180, VLOCL0804; Covidien), we closed the post-ESD ulcer ( Fig. 1 ). Procedure: Suturing followed a previously described colorectal method 2 . The right-channel holder grasped the needle to initiate EHS at the right and proximal edges of the defect. The first bite was placed distally, and continuous lateral suturing was performed from right to left ( Fig. 2 ). Each time the needle emerged, it was immediately secured by the left channel holder. The right holder was released, the left holder was rotated to withdraw the needle ( Fig. 3 ), and an in-air handoff returned the needle to the right holder to continue the running suture ( Fig. 4 ). The suture line was tightened using a holder to achieve complete defect closure ( Fig. 5 ). No delayed bleeding has occurred to date. This two-holder EHS enables safer, more efficient closure after ESD and may expand its application in advanced endoscopic therapy.

Complete closure of a post-ESD ulcer using two-holder endoscopic hand suturing for precise and stable mucosal defect closure. ESD, endoscopic submucosal dissection.Video 1

Endoscopy_UCTN_Code_TTT_1AQ_2AK
